# A Time-Frequency Measurement and Evaluation Approach for Body Channel Characteristics in Galvanic Coupling Intrabody Communication

**DOI:** 10.3390/s21020348

**Published:** 2021-01-06

**Authors:** Ziliang Wei, Yangrong Wen, Yueming Gao, Mingjing Yang, Jiejie Yang, Sio Hang Pun, Mang I Vai, Min Du

**Affiliations:** 1 College of Physics and Information Engineering, Fuzhou University, Fuzhou 350108, China; N181127039@fzu.edu.cn (Z.W.); N191127040@fzu.edu.cn (Y.W.); yangmj5@fzu.edu.cn (M.Y.); chy@fzu.edu.cn (J.Y.); W1701@fzu.edu.cn (M.D.); 2 Key Lab of Medical Instrumentation & Pharmaceutical Technology of Fujian Province, Fuzhou 350108, China; 3 State Key Laboratory of Analog and Mixed-Signal VLSI, University of Macau, Macau SAR, Macau 999078, China; lodgepun@um.edu.mo (S.H.P.); fstmiv@um.edu.mo (M.IV.); 4 Department of Electrical and Computer Engineering, Faculty of Science and Technology, University of Macau, Macau 999078, China

**Keywords:** intrabody communication, parallel measurement, multitone excitation, group search algorithm, time-frequency evaluation

## Abstract

Intrabody communication (IBC) can achieve better power efficiency and higher levels of security than other traditional wireless communication technologies. Currently, the majority of research on the body channel characteristics of galvanic coupling IBC are motionless and have only been evaluated in the frequency domain. Given the long measuring times of traditional methods, the access to dynamic variations and the simultaneous evaluation of the time-frequency domain remains a challenge for dynamic body channels such as the cardiac channel. To address this challenge, we proposed a parallel measurement methodology with a multi-tone strategy and a time-parameter processing approach to obtain a time-frequency evaluation for dynamic body channels. A group search algorithm has been performed to optimize the crest factor of multitone excitation in the time domain. To validate the proposed methods, in vivo experiments, with both dynamic and motionless conditions were measured using the traditional method and the proposed method. The results indicate that the proposed method is more time efficient ($T_{meas}=1\;\mathrm{ms}$) with a consistent performance ($\rho_{c}$ > 98%). Most importantly, it is capable of capturing dynamic variations in the body channel and provides a more comprehensive evaluation and richer information for the study of IBC.

## 1. Introduction

Galvanic coupling intrabody communication (IBC) is a low-power, low-cost wireless communication scheme that utilizes the human body as a medium to develop a body area network in IEEE 802.15.6 [1,2]. Currently, in measuring the channels of IBC, most research studies assume that the channels within the body are motionless and neglect all dynamic variations within. Obviously, such assumption is merely applicable to limited scenarios in a biological system. For instance, in the studies of leadless cardiac pacemakers, motionless measurements can only obtain valid channel characteristics of the cardiac system at a certain state during systole to diastole [3,4,5]. In addition, people’s daily activities will inevitably lead to dynamic body channels. Therefore, evaluating dynamic body channels is required and a novel measuring approach that can reflect dynamic body channel characteristics needs to be developed. In order to deal with the dynamic body channel characteristics, short measuring time is indispensable for accurate measurement.

**Figure 1:**
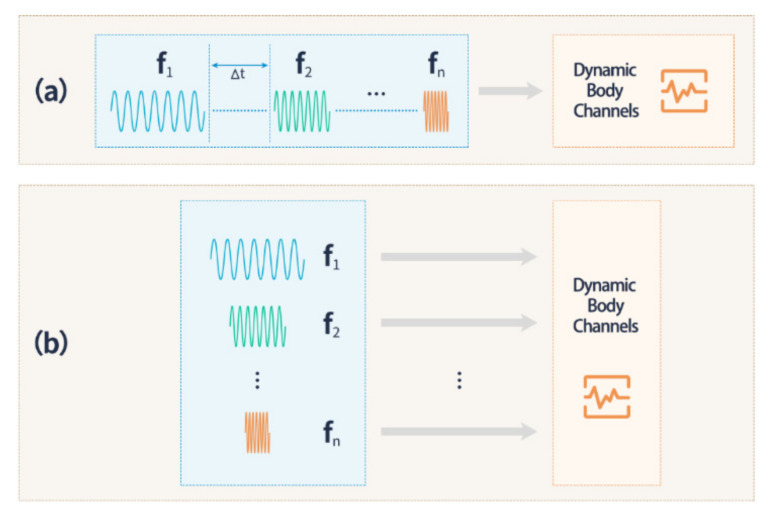
(**a**) Traditional sequential measuring scheme. (**b**) A novel parallel measuring strategy. ∆t represents the time interval when sweeping different frequencies, called the temporal resolution, and ∆t = 0 in the parallel measuring strategy.

To date, modeling and measurement of motionless body channel characteristics have been extensively studied [6,7,8,9], while few research studies on dynamic channels have been reported [10,11]. In the studies of motionless measurements, single-frequency measurements or frequency-sweep operations are mostly employed, hereinafter referred to as traditional methods. Single-frequency measurements are mostly performed using a signal generator combined with an oscilloscope [12,13,14,15,16]. Where available, frequency sweep measurements are typically carried out using a high-precision instrument such as a spectrum analyzer [17,18,19]. However, measuring time has rarely been considered in the traditional methods. As shown in Figure 1a, the total measuring time for traditional methods includes the measuring time for each frequency and the interval time (∆t) between the frequencies. The total time can be much longer than the body channel variations because of the cardiac or other activities. Therefore, the traditional schemes do not represent a comprehensive tool to evaluate many dynamic body channels. On the other hand, researchers have explored the differences between the human body in dynamic and motionless situations. For example, Schenk et al. [20] investigated the channel characteristics when the human body is walking, standing, and moving the arm up and down versus sitting in a stationary position. Wegmuller et al. [21,22] extensively explored the variations in the effect of galvanic coupling IBC during human activities. Callejon et al. [12] also discussed the influences of human motion on capacitive coupling versus galvanic coupling IBC.

The most important reason why traditional methods cannot comprehensively evaluate dynamic body channels is that their long measuring time does not satisfy the time requirements of dynamic body channels. If traditional methods are considered as a “sequential” measurement, then a corresponding “parallel” measurement could be considered as depicted in Figure 1b. Thus, we believe that a parallel measurement can greatly reduce the measuring time in order to fulfill the time requirement of dynamic body channels. In this work, we present a parallel measuring scheme that can excite multiple measuring frequencies simultaneously to greatly reduce the measuring time. We concentrate the measuring frequencies on a multitone excitation, which is conducted by inverse fast Fourier transform (IFFT). At the receiving end, a corresponding FFT operation is performed to recover each frequency component, which enables a “parallel” measuring strategy.

This technique can compensate for the weakness and inadequacy of traditional approaches for the analysis of dynamic body channels and provides a more rigorous evaluation of dynamic biosystems. The rest of this paper is organized as follows. In Section 2, the design principles of multitone excitation are presented and the optimization of the method is discussed. Section 3 describes the specific implementation of multitone excitation and the actual measurement of a quasi-periodic channel via in vivo experiments. Section 4 provides a comprehensive analysis and comparison of the experimental results and proposes the time-parameter approach. Section 5 and Section 6 discuss and conclude the work in this paper, respectively.

## 2. Multitone with Optimization

A Multitone excitation is a periodic broadband signal that is composed of a superposition of sinusoidal signals at different frequencies. With limited energy applied, the discrete spectrum of the multitone excitation reaches the maximum signal-to-noise ratio at the exciting frequencies. Multitone excitation dramatically reduces the measuring time while preserving the advantages of sine waves as much as possible. Its mathematical expression ($e\left(t\right)$) can be represented as:

(1) $$e(t)=\sum_{n=1}^{N}A_{n}\cos\left(2\pi f_{n}t+\varphi_{n}\right)$$

where *N* is the number of excitation frequencies; *A_n_*, *f_n_* and *φ_n_* are the amplitude, frequency and phase of the nth excitation frequency.

According to Equation (1), the multitone excitation is similar to the formulation of inverse Fourier transform (IFT). Therefore, multitone excitation can be implemented in LabVIEW using the IFFT approach. However, since multitone excitation contains rich frequencies, once the peaks at different excitation frequencies are accumulated, the peak-to-average ratio of multitone excitation will be too high. To reflect the qualities and performances of multitoned excitation in the time domain, the crest factor is designed and is defined as

(2) $$CF(e)=\frac{l_{\infty }(e)}{rms(e)}=\frac{\max_{t\in [0,T]}|e(t)|}{\sqrt{\frac{1}{T}\int_{0}^{T}{\left|e(t)\right|}^{2}dt}}$$

where *l_∞_*(*e*) is the maximum value of the continuous signal *e*(*t*) in the range [0, *T*], also known as the Chebyshev norm; *rms*(*e*) denotes the root mean square value of the signal. In most situations, low crest factors of the excitation and response signals are desirable because this maximizes the signal-to-noise ratio (SNR) for a given range of signal allowable amplitudes.

**Figure 2:**
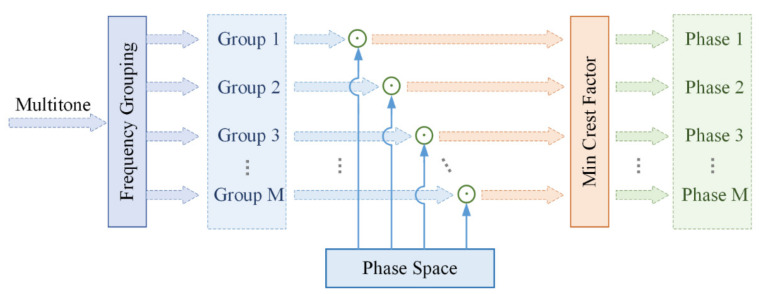
Flow diagram of the grouping search algorithm.

To minimize the crest factor, the phase of individual excitation frequency *φ_n_* can be adjusted by our proposed group search algorithm. This algorithm based on the principle of the partial transmission sequence technique, which is commonly used in Orthogonal Frequency Division Multiplexing (OFDM) systems, to reduce the peak-to-average ratio (PAPR), as shown in Figure 2.

The specific steps are listed as follows.

- All frequencies in the frequency distribution are equally spaced into M groups.
- A phase factor space W that is flexible and variable in phase is set.
- The M groups, respectively, perform a traversal search of the phase in the phase factor space W.
- The crest factor for each combination approach is calculated, and the minimum values are screened out.
- The phase combination scheme corresponding to the minimum value of the crest factor is returned.

**Figure 3:**
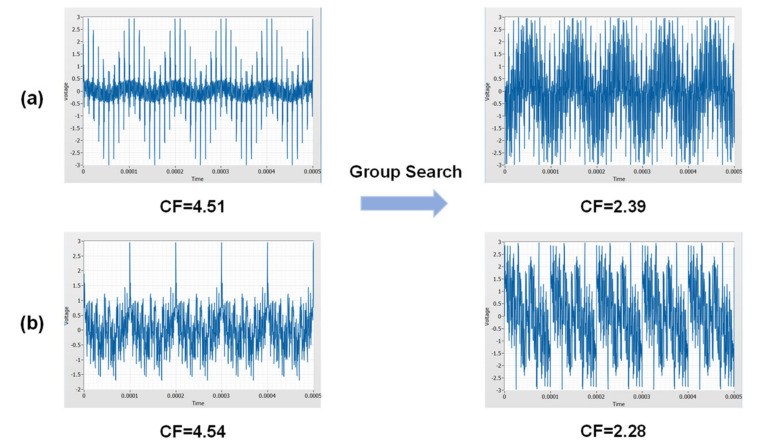
The time domain waveform and its crest factors (CF) of the multitone before and after group search algorithm. (**a**) denotes linearly distributed multitone and (**b**) denotes quasi-logarithmically distributed multitone.

The excitation frequencies in this work ranged from 10 kHz to about 1 MHz was linearly and quasi-logarithmically distributed, as shown in Table 1. The larger the number of phase factors in the phase space, the more calculations are required for the group search algorithm. The algorithm achieves a satisfactory result when the number of phase factors reaches four. Therefore, the phase space in this work is set as [0, 0.5π, π, 1.5π]. Figure 3 shows the time domain waveform and its crest factors of two multitoned excitations under liner and quasi-logarithmical distribution before and after the group search algorithm. Evidently, both distributions are optimized to have significantly lower crest factors.

## 3. Experiments

### 3.1. Materials and Settings

**Figure 4:**
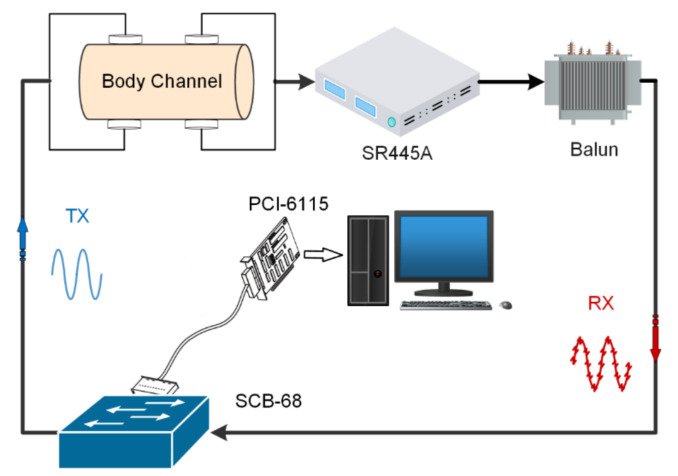
Schematic diagram of experimental platform construction.

To test and validate the performance of the proposed parallel measurement for dynamic body channel characterization, a multitone excitation measurement platform was built (shown in Figure 4). A computer equipped with a multifunction high-speed digital I/O card (PCI-6115, National Instruments, Austin, TX, USA) (10 MS/s/ch, 12 bit) with its matching terminal block SCB-68, was used to generate the multitone excitation and receive the responses from the body channel via a signal amplifier (SR445A, Stanford Research Systems, Sunnyvale, CA, USA). Additionally, a balun transformer (FTB-1-6*A15+ (50 Ω), Mini-Circuits, New York City, NY, USA) was used to break the ground loop between the transport (TX) and receive (RX) of the measurement.

The PCI-6115 is a versatile data acquisition (DAQ) device with synchronous sampling, which is connected to the main board of the computer through the PCI interface. It provides analog I/O, digital I/O, two 24-bit counters, and a digital trigger. It has a maximum sampling rate of 10 MS/s per channel and includes an analog-to-digital converter (ADC) with 12-bit resolution. The other end of the PCI-6115 is connected to the matching terminal block SCB-68 via a shielded cable SH68-68-EPM (National Instruments, Austin, TX, USA). The SCB-68 is a junction box and transfer station. The output and acquisition of signals are controlled by LabVIEW and PCI-6115, and are relayed in the SCB-68. Differential wiring is applied at the receiving end for data acquisition.

During the experiment, we recruited a volunteer (male, 24 years old, 172 cm, 58 kg) to participate and his forearm was selected as the measurement site. A four electrode (physiotherapy electrodes, 24 mm × 24 mm, Shanghai Trans Health Biotechnology Co., Ltd., Shanghai, China) arrangement, which formed a typical galvanic coupling IBC configuration, was made for testing the proposed parallel multitone excitation method.

### 3.2. Calibration

Measurement systems inevitably have some inherent inaccuracies, such as errors caused by the components, environmental variations, and systematic drifts, etc. To minimize these errors, a calibration was performed prior to the experiment. Before the measurement, the TX electrodes were connected directly to the RX electrodes to perform a complete measurement process. The resultant was used to offset the error for the rest of the measurements.

### 3.3. Measurements

**Figure 5:**
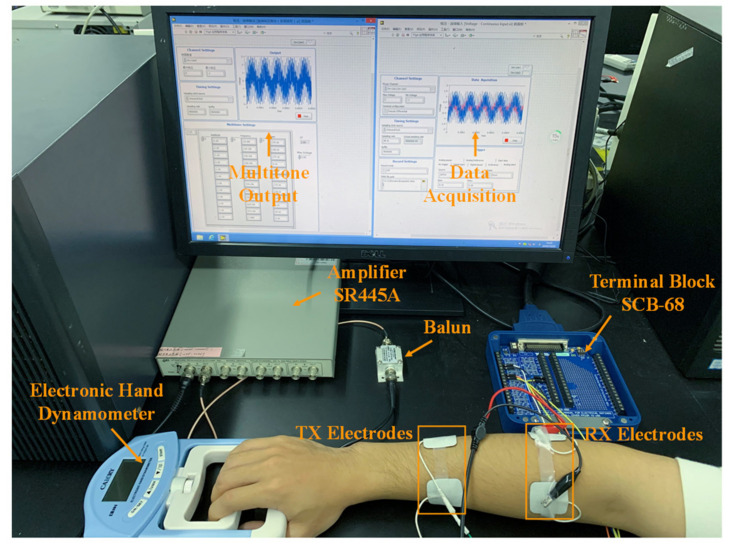
Measurement configuration for measuring motionless and dynamic body channel characteristics.

A photo of the in vivo measurement setup is shown in Figure 5. The body channel length (the distance between the center of the TX electrodes and the center of the RX electrodes) on the forearm of the subject was 10 cm. During the motionless measurement, the volunteer remained seated and rested his forearms still and flat on the table. Both excitations with linear and quasi-logarithmic frequency distributions were then measured consecutively using traditional single-frequency measurement methods. Each excitation was measured three times under the same conditions and averaged. Then, the measurements were repeated with parallel measurements with the multitone strategy.

Then, similar measurements were performed for the dynamic body channel measurement. To mimic a dynamic body channel, an electronic hand dynamometer (model: EH101, Zhongshan Camry Electronic Co., Ltd., Zhongshan, China) was used. During an approximately 8 s measurement period, the volunteer was requested to perform the grip strength test a different number of times (one, two or three) using the dynamometer. This served as controlled dynamic body channel variations and the variations can be identified for validating the effectiveness of the proposed measurement methods. The volunteer was requested to maintain the grip strength uniformly during the experiments and each grip strength was about between 30 kg and 33 kg. Then, the parallel measurement was performed under the same requirements and conditions as the motionless measurement with both linear and quasi-logarithmic frequency distributions.

## 4. Results

When evaluating body channel characteristics in the frequency domain, the traditional method gives direct results at each excitation frequency. Multitone excitation, on the other hand, is a mixed-signal process that requires the separation of each excitation frequency by the FFT operation. Since NI-LabVIEW is used for data acquisition, it is convenient to use the signal analysis tools in LabVIEW to process the data during signal processing. In the multitone strategy, the acquired data are used to calculate the amplitude at different excitation frequencies by the FFT processing tool in LabVIEW, and then compared with the transmitted signal to obtain the attenuation. In this work, in all motionless cases where the multitone strategy was used to evaluate body channel characteristics in the frequency domain, the FFT operations were performed on the 1 ms (10 multitone cycles) measurement data.

In addition, compared to traditional methods, the multitone strategy may give rise to noise and interference in that the energy at the excitation frequencies may leak to other frequencies. To avoid this spectral leakage as much as possible, the signal must be windowed before the FFT operation. From the viewpoint of reducing spectrum leakage, the Hanning window is superior to most window functions. However, the main lobe of the Hanning window is wider, which is equivalent to a wider analysis bandwidth and lower frequency resolution. While the excitation frequency interval in this work is relatively large, and the frequency resolution requirement is moderate, so the Hanning window is a decent choice. The windowing operations were also performed in LabVIEW.

To demonstrate the equivalence of the results obtained by different methods, the concordance correlation coefficient $\rho_{c}$ was calculated, which is defined as

(3) $$\rho_{c}=\frac{2\rho \sigma_{x}\sigma_{y}}{\sigma_{x}^{2}+\sigma_{y}^{2}+{\left(\mu_{x}-\mu_{y}\right)}^{2}}$$

where $\mu_{x}$ and $\mu_{y}$ are the means of the two variables and $\sigma_{x}^{2}$ and $\sigma_{y}^{2}$ are the corresponding variances. ρ is the correlation coefficient between the two variables. All of the attenuation curves in dB were obtained as the ratio between the received and the transmitted voltage, according to

(4) $$\mathrm{Attenuation}\;(\mathrm{dB})=20\log_{10}\left(\frac{V_{\mathrm{RX}}}{V_{\mathrm{TX}}}\right)$$

### 4.1. System Results

**Figure 6:**
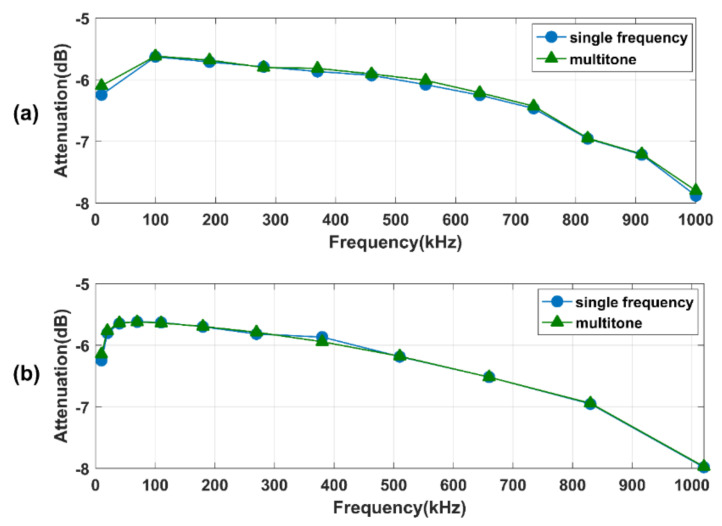
The calibration results of the measurement system for linear (**a**) and quasi-logarithmical (**b**) distributions.

Figure 6 shows the calibration results of the measurement system for two different frequency distributions. The blue curve shows the average calibration results for all the excitation frequencies in Table 1 using the single-frequency method. The green curve shows the average calibration results of parallel measurements using the multitone strategy. As shown in the figure, the calibration of the system first rises briefly in the range of 10 kHz to 100 kHz and then declines steadily from about 100 kHz onwards. This is mainly due to the design of the high-speed digital card. Additionally, the concordance correlation coefficient between the results obtained using the single-frequency approach and the multitone strategy are 99.6% and 99.8% for linear and quasi-logarithmic distributions. Therefore, it can be confidently stated that the multitone strategy is equivalent to the traditional single-frequency method.

**Figure 7:**
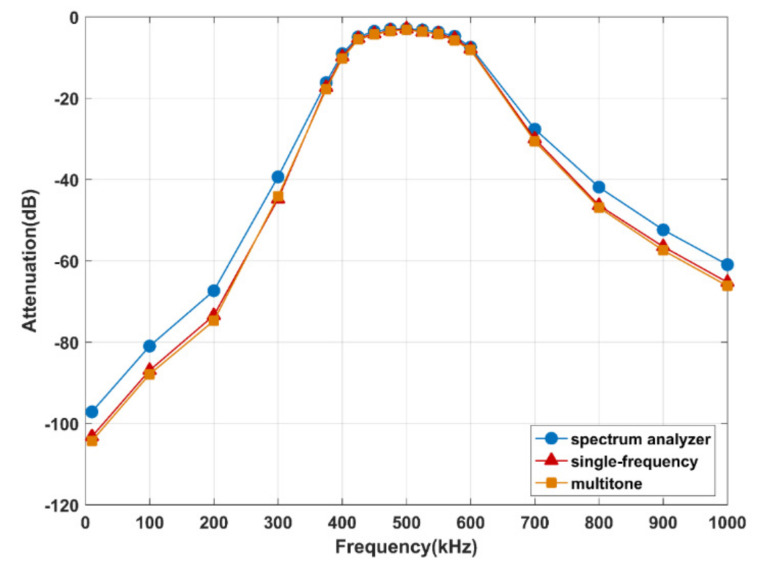
Results of the standard filter measurements using a spectrum analyzer (blue) and the system in this paper (red and orange), respectively.

Figure 7 presents the results of the standard filter (center frequency 500 kHz, bandwidth 100 kHz, matching impedance 50 Ω) measurements using a spectrum analyzer (CXA N900A, Agilent Technologies Inc., Santa Clara, CA, USA) (blue) against the measurement system designed in this manuscript (red and orange). For measurements with attenuation above −40 dB, the concordance correlation coefficient between multitone and spectrum analyzer is 98.7%, which demonstrates that the accuracy of the measurement system in this paper is nearly identical to that of the spectrum analyzer in that range. Regarding the experiment preformed in this research, the attenuation was generally above −40 dB. Therefore, the experimental system proposed in this article is suitable and accurate enough. On the other hand, Figure 7 also shows that the measurements with the single frequency method and multi-tone method are very close. The concordance correlation coefficient between the results of the two methods is 99.9%, which also indicates that the multitone excitation strategy is equivalent to the traditional single-frequency measurements.

### 4.2. Motionless Results

**Figure 8:**
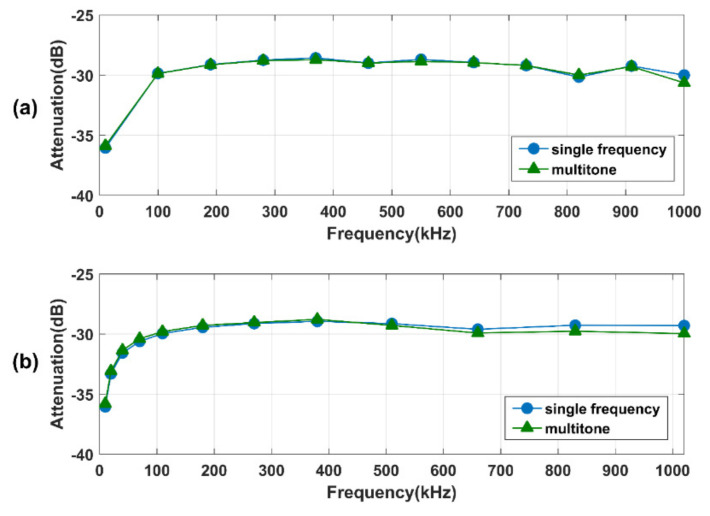
The results of the motionless body channel characteristics under linear (**a**) and quasi-logarithmic (**b**) distributions.

Figure 8 shows the results of the motionless body channel characteristics of excitation with linear and quasi-logarithmic frequency distributions. The blue curves represent the characteristics obtained with the traditional single-frequency method, and the green curves represent the characteristics obtained with the multitone excitation method. The trend of the measurements is consistent regardless of the excitation methods. The concordance correlation coefficient between the single-frequency measurement and the multitone strategy are 99.4% and 98.8% for linear and quasi-logarithmic distributions. The two approaches exhibit larger attenuation in the exciting frequency below 100 kHz, which is consistent with the results of many related investigations, mainly due to the high resistant of stratum corneum at low frequencies [23]. The results finally demonstrate that the multitone excitation strategy can achieve nearly the same performance ($\rho_{c}$ > 98%) as the traditional method.

More importantly, in terms of measuring time, the multitone strategy spent only 1 ms, which will be specifically calculated in the next section. The time spent on single-frequency measurements is difficult to calculate in specifics, but it is at least measured in seconds. The frequency sweeping method with a spectrum analyzer, for example, would take 1.2 s over the same frequency range with a resolution bandwidth of 1 kHz. This is the main reason why traditional methods cannot comprehensively evaluate the dynamic body channel. Take the cardiac channel as an example, the average cardiac cycle of an adult is 0.88 s. Within this 0.88 s, traditional methods cannot even perform a complete measurement, but only obtain the channel characteristics of the cardiac in a certain state. The multitone strategy can complete 880 measurements in the same amount of time, which has enough time resolution to capture the dynamic variations.

### 4.3. Dynamic Results

#### 4.3.1. Measurement of the Proposed Method

This method differs from traditional methods by having higher sensitivity in measuring time domain parameters, which is so called the time-parameter approach. The frequencies chosen in our work are all integer multiples of 10 kHz, while the period of the multitone excitation is the smallest common multiple of all excitation frequencies. Thus, all the periods of the multitone excitation are *T_m_* = 0.1 ms. Then, the sampling rate is *fs* = 4 MS/s, and the acquisition duration is *T_total_* = 8 s. Accordingly, a number of *N_total_* = *T_total_*/*T_m_* = 80,000 cycles are required for multitone excitation. During signal processing phase and FFT transformation, n multitone cycles are taken as one complete measurement. Thus, a complete measuring time is *T_meas_* = *n* × *T_m_* (*n* ∈ *N*). Integer multiple cycles avoid spectrum leakage in the frequency domain as much as possible. For example, if 10 multitone cycles (*n* = 10) are taken as one complete measurement, it only takes 1 ms to complete a parallel measurement of multiple frequencies. This means that we have taken 8000 complete measurements in 8 s at once, and the time resolution for resolving the dynamic variations is 1 ms. As a result, the multitone excitation strategy is more time efficient, which allows us to obtain the dynamically varying features of the channel with *T_meas_* time resolution.

#### 4.3.2. Dynamic Characteristics

**Figure 9:**
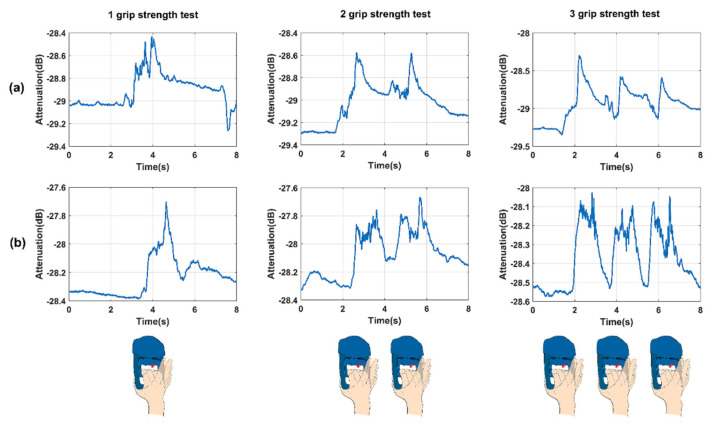
The dynamic body channel characteristics of the volunteers performing 1, 2, and 3 grip strength tests under (**a**) 550 kHz in linear distribution and (**b**) 510 kHz in quasi-logarithmic distribution.

According to the previous analysis, the measuring time were taken 100 multitone (*n* = 100) cycles as one complete measurement. Then, the total time 8 s can be divided into 800 complete measurements with 10 ms time resolution. Thus, it is convenient to obtain the variable characteristics of dynamic body channels. Figure 9 shows the dynamic body channel characteristics of the volunteer performing one, two, and three grip strength tests under 550 kHz in a linear distribution and 510 kHz in a quasi-logarithmic distribution. The dynamic features of channel variations of the forearm during movement were captured and are shown in the Figure 9. The number of grip strength tests done by the volunteer has a corresponding number of abrupt changes in the dynamic characteristics of the channel. This result indicates that the multitone strategy with the time-parameter approach successfully extracts variations in the dynamic body channel by the homemade instruments.

#### 4.3.3. Time-Frequency Evaluation

**Figure 10:**
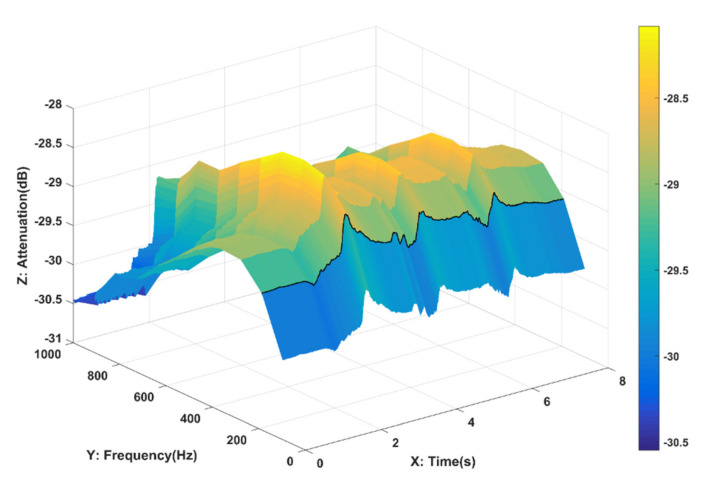
The time-frequency domain characteristics of the body channel during 3 grip strength tests performed by the volunteer during an 8 s period with linear frequency distribution method.

Based on the multitone strategy, a time-frequency domain analysis for evaluating dynamic body channels is further proposed. Different from traditional evaluation methods, this analysis is able to reflect both the time-domain and frequency-domain characteristics of the dynamic body channel at a given time. Figure 10 shows the time-frequency domain characteristics of the body channel during three grip strength tests performed by the volunteer during an 8 s period. XZ axes show the variations in the dynamic body channel at each frequency for a specific period of time. YZ axes shows the motionless body channel characteristics similar to those of the traditional method. In the case of dynamic body channels, this approach provides a more comprehensive evaluation and richer information.

## 5. Discussion

Currently, most researchers are exploring the sensitivity of IBC to human motion using traditional evaluation methods while neglecting the specific variations in the dynamic body channel. The proposed multitone excitation strategy achieves a more comprehensive evaluation of dynamic body channels than the traditional method. This approach compensates for the inadequacy of traditional evaluation methods in dynamic body channels. The multitone excitation strategy has less restriction on the interval time (∆t = 0), which eliminates the need to make time-invariant assumptions about the system during measurements. Thus, this exclusion is more rigorous for dynamic biosystems.

However, the trade-off that must be paid for applying this novel approach is the increased complexity of the multitone implementation and processing. For example, additional IFFT operations are required to achieve parallel measurements, and additional crest factor reduction operations are required to ameliorate multitone time domain defects. In addition, the sampling rate is only twice that of the Nyquist frequency due to the limitations of the on-board hardware acquisition equipment and its memory, and compared to the spectrum analyzer, the high-speed digital card in this experiment has a lower anti-noise performance and small signal resolution. This condition may result in some loss of accuracy in some excitation frequencies. When future experimental conditions permit, a high-speed digital card with a higher sampling rate and higher accuracy will be used for repeated experiments.

The parallel measurement approach in our work has achieved satisfactory results in dynamic body channels. Thus, its origin technique (OFDM) may also be capable of excellent application in many dynamic body channels. For instance, when OFDM technology is adopted for communication in the intracardiac communication technology of wireless pacemakers, it can cope well with the frequency selective fading of the channel, owing to the beating of the heart. The adoption of OFDM technology can also greatly improve the bandwidth efficiency due to the limited frequency band resources for galvanic coupling IBC.

## 6. Conclusions

In this work, a parallel measuring strategy incorporating time parameters approach is used to achieve a time-frequency evaluation for body channel characteristics, which provides a more time efficient strategy and a better understanding than the traditional scheme. The major contributions of this study are summarized as follows.

(1) A multitone excitation measuring strategy is designed and implemented to achieve parallel measurements for body channels.
(2) A grouping search algorithm has been performed to reduce the crest factor of multitone excitation in the time domain.
(3) A time parameter processing approach is proposed to capture the dynamic body channel characteristics and a time-frequency evaluation is further proposed.

The experimental results show that the multitone excitation strategy can achieve the same performance ($\rho_{c}$ > 98%) as traditional methods with a higher time efficiency ($T_{meas}=1\;\mathrm{ms}$) and it can successfully capture variations in the dynamic body channel by the homemade instruments. In the case of dynamic body channels, time-frequency approach provides a more comprehensive evaluation and richer information.

Furthermore, this novel approach may be used to investigate the intracardiac communication for leadless pacemakers and the influence of gastrointestinal motility on the communication quality of endoscopic capsule. In the near future, the capabilities of parallel measurement and the time parameter approach can provide more medical information and communication applications for motionless and dynamic body channels.

## Figures and Tables

**Table 1:** The excitation frequencies in this work ranged from 10 kHz to about 1 MHz.

Frequency Distribution	1	2	3	4	5	6	7	8	9	10	11	12
Linear (kHz)	10	100	190	280	370	460	550	640	730	820	910	1000
Quasi-log (kHz)	10	20	40	70	110	180	270	380	510	660	830	1020

## Data Availability

The data presented in this study are available on request from the corresponding author. The data are not publicly available due to privacy.
